# Rhesus macaques vaccinated with consensus envelopes elicit partially protective immune responses against SHIV SF162p4 challenge

**DOI:** 10.1186/1743-422X-10-102

**Published:** 2013-04-02

**Authors:** Hermancia S Eugene, Brooke R Pierce-Paul, Jodi K Cragio, Ted M Ross

**Affiliations:** 1 Center for Vaccine Research, University of Pittsburgh, 9047 BST3; 3501 Fifth Avenue, Pittsburgh, PA, 15261, USA; 2 Department of Microbiology and Molecular Genetics, University of Pittsburgh, Pittsburgh, PA, USA; 3 Graduate Program in Molecular Virology and Microbiology, University of Pittsburgh, Pittsburgh, PA, USA

## Abstract

The development of a preventative HIV/AIDS vaccine is challenging due to the diversity of viral genome sequences, especially in the viral envelope (Env_160_). Since it is not possible to directly match the vaccine strain to the vast number of circulating HIV-1 strains, it is necessary to develop an HIV-1 vaccine that can protect against a heterologous viral challenge. Previous studies from our group demonstrated that a mixture of wild type clade B Env_gp160s_ were able to protect against a heterologous clade B challenge more effectively than a consensus clade B Env_gp160_ vaccine. In order to broaden the immune response to other clades of HIV, in this study rhesus macaques were vaccinated with a polyvalent mixture of purified HIV-1 trimerized consensus Env_gp140_ proteins representing clades A, B, C, and E. The elicited immune responses were compared to a single consensus Env_gp140_ representing all isolates in group M (Con M). Both vaccines elicited anti- Env_gp140_ IgG antibodies that bound an equal number of HIV-1 Env_gp160_ proteins representing clades A, B and C. In addition, both vaccines elicited antibodies that neutralized the HIV-1_SF162_ isolate. However, the vaccinated monkeys were not protected against SHIV_SF162p4_ challenge. These results indicate that consensus Env_gp160_ vaccines, administered as purified Env_gp140_ trimers, elicit antibodies that bind to Env_gp160s_ from strains representing multiple clades of HIV-1, but these vaccines did not protect against heterologous SHIV challenge.

## Introduction

One of the greatest struggles for developing a preventative human immunodeficiency virus (HIV)/acquired immunodeficiency syndrome (AIDS) vaccine is overcoming the diversity of viral isolates [1]. The Env_gp160_ sequences can differ up to 35% between clades and ~15% within a specific clade [2]. Viruses classified as clade B are responsible for ≥40% of infections in the Americas and Europe, but in Asia and sub-Saharan Africa, where most new infections are recorded each year, other clades are dominant. Most new infections in these regions are classified as clades A, C, or A/E viruses [1,3]. Any HIV vaccine that will prevent infection must be able to overcome the diversity of HIV sequences.

To overcome the HIV sequence diversity, polyvalent mixture of antigens and consensus proteins were designed [4-7]. Polyvalent vaccines increase breadth by including multiple copies of a target (s) or epitopes into a single formulation. Polyvalent vaccine strategies have been employed to increase the breadth of the humoral and cellular immune responses [8,9]. Polyvalent mixtures of Env_gp140_ or HIV proteins (Gag-Pol, Tat and trimeric Env_gp140_) elicit a degree of protection against heterologous challenge [8,10]. Consensus-based vaccines rely on a centralized antigen designed to reduce sequence diversity by using the most common amino acid at each position of the protein. Consensus vaccines are designed to reduce the genetic differences between the vaccine and the primary isolate and increase the breadth of immune responses [11-14].

To overcome the diversity in Env_gp160_ sequences and to design a more effective AIDS vaccine, consensus Env_gp140_ sequences were designed for 4 clades of HIV-1 (A, B, C, and E), as well as a single consensus Env_gp160_ representing isolates from all of Group M. For the first time, in the same study, consensus A, B, C, and E Env_gp140_ sequences were used in a polyvalent vaccine mixture, and compared to a Con M Env_gp160_, to assess the ability to elicit a broadly reactive anti-Env_gp160_ immune response. The immunological responses of the polyvalent mixture in vaccinated rhesus macaques were compared to that of the single Con M Env_gp140_ vaccine. Both vaccines elicited anti-Env immune responses against multiple clades of HIV; however neither vaccine strategy efficiently protected monkeys against a SHIV_SF162p4_ challenge.

## Results

### Characterization of consensus envelopes

The goal of this study was to design a HIV Env_gp160_ vaccine that elicits broadly reactive immune responses in an effort to overcome the inherent diversity in the Env_gp160_. Therefore, an HIV-1 group M consensus Env_gp140_ vaccine was compared to a polyvalent mixture of clade consensus Env_gp140s_ representing 4 individual clades of HIV-1 (A, B, C, and E). The *env* gene sequences were then truncated at the transmembrane domain, and the cleavage site mutated, to generate a Env_gp140_[15]. To stabilize the truncated Env_gp140_ trimers, the bacteriophage fibronectin domain (FT) was added to the 3’ end of the Env_gp140_ sequence, as previously described [15].

**Figure 1:**
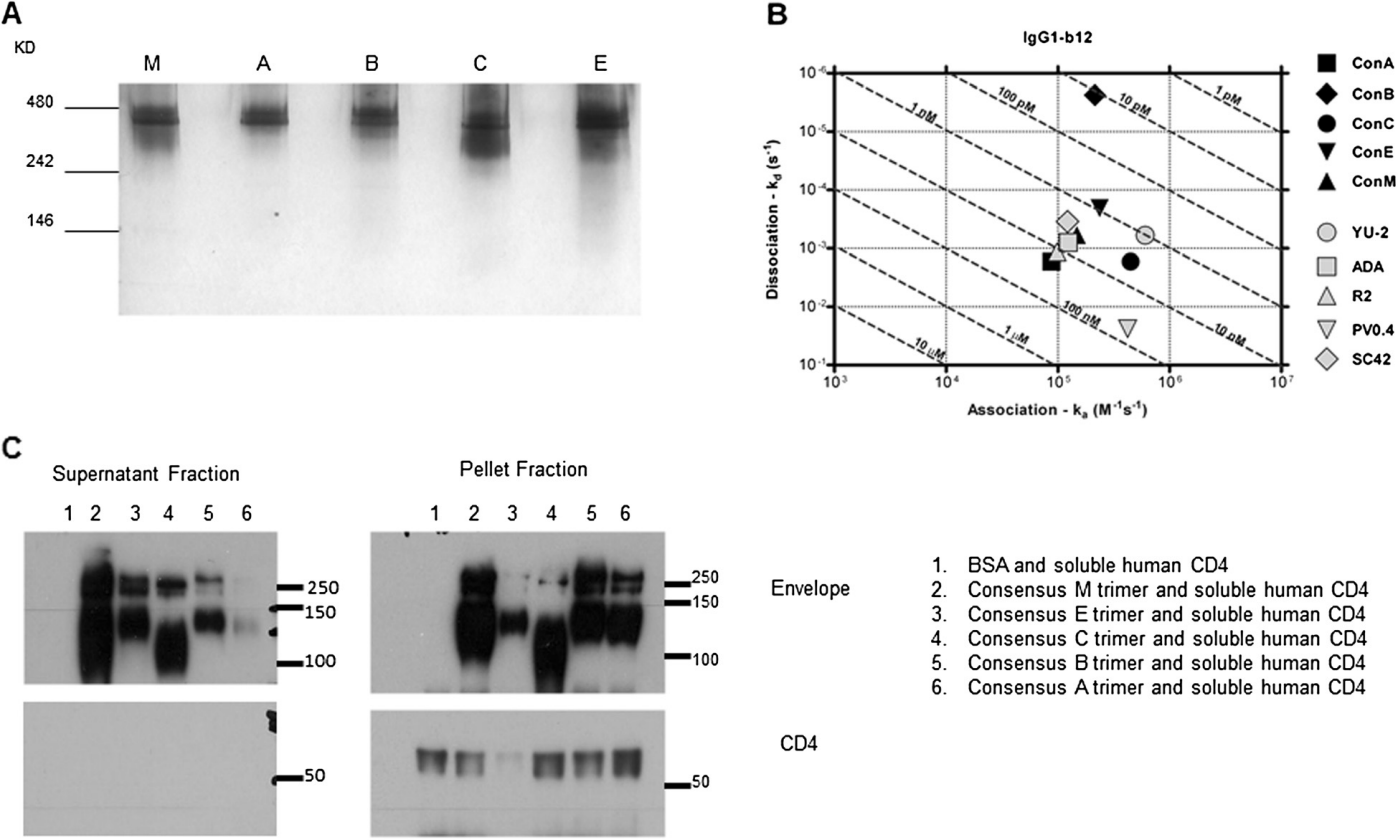
**Characterization of consensus Env**_**gp140s**_**. A)** 1 μg of each purified consensus Env_gp140_ was loaded onto a native PAGE and then sliver stained. The top of the gel is labeled with the consensus Env_gp140_ present in the lane and the protein ladder values are present on the y-axis. **B)** Using surface plasmon resonance, the interaction of the consensus Env_gp160s_ and b12 was investigated in solution. The x-axis gives the rates of association between b12 and the Env_gp140_ and the y-axis gives the dissociation between the Env_gp160s_ and b12. The consensus trimers are indicated by the darker symbols on the graph and the primary envelope trimers are indicated by the lighter symbols. **C)** The consensus Env_gp140s_ and negative control BSA was pre-incubated with Histag soluble human CD4 at 37°C then mixed in with magnetic beads that were pre-incubated with the anti-his antibody. Using immunoprecipitation (IP), sCD4 and anything bound to it was pulled down in the pellet fraction. Supernatants and pellet fraction was analyzed by SDS-PAGE and western blot using anti-his and anti- Env_gp160_ antibodies. Left panel: supernatants fraction. Right panel: pellet fraction (sCD4 and proteins bound). The protein ladder values on y-axis. The upper blots were probed with rabbit polyclonal anti- Env_gp160_ antibody and the bottom blots were probed with mouse anti-human CD4 antibody (Clone RFT-4g mouse IgG, SouthernBiotech). Lane 1: BSA, Lane 2: Con M Env_gp140_, Lane 3: Consensus E Env_gp140_, Lane 4: Consensus C Env_gp140_, Lane 5: Consensus B Env_gp140_, Lane 6: Consensus A Env_gp140_.

Purified trimerized Envs were detected at ~480kDa size indicating oligomerization as trimer proteins (Figure 1A). Some Env dimers were observed in consensus C, E and M Env_gp140_ protein fractions. To probe the antigenic structure, the broadly reactive monoclonal antibody b12 [16] was used to determine binding kinetics to each consensus envelope by surface plasmon resonance (SPR) on a Biacore 3000 (Figure 1B and Additional file 1: Figure S1). The rate of association between the consensus Env_gp140_ trimers and b12 was similar to the rate of association between b12 and primary Env_gp140_ trimers. The rate of dissociation of b12 from all the Env_gp140_ trimers was similar, except for consensus B, which had a slower rate of disassociation. Each Env_gp140_ bound to the primary HIV receptor, human CD4 (hCD4) (Figure 1C). The MAb b12 is a monoclonal isolated from an infected patient with a consensus B wild-type Env_gp160_ on its viral surfaces and therefore may recognize clade B Env_gp160_ with more efficiency than non-clade B Env_gp160s_. In addition, consensus B envelopes are bound with higher affinity antibodies to clade B Env_gp160s_, but less so with consensus C Env_gp140_. The reverse is true as well. A polyclonal serum, HIV-Ig, was obtained from the AIDS Reference and Reagent Program. This polyclonal serum, collected from a clade B infected person, may not recognize the consensus E Env_gp160_ with the same affinity and efficiency as the consensus B Env_gp160_.

### Vaccination of non-human primates with consensus envelopes

**Figure 2:**
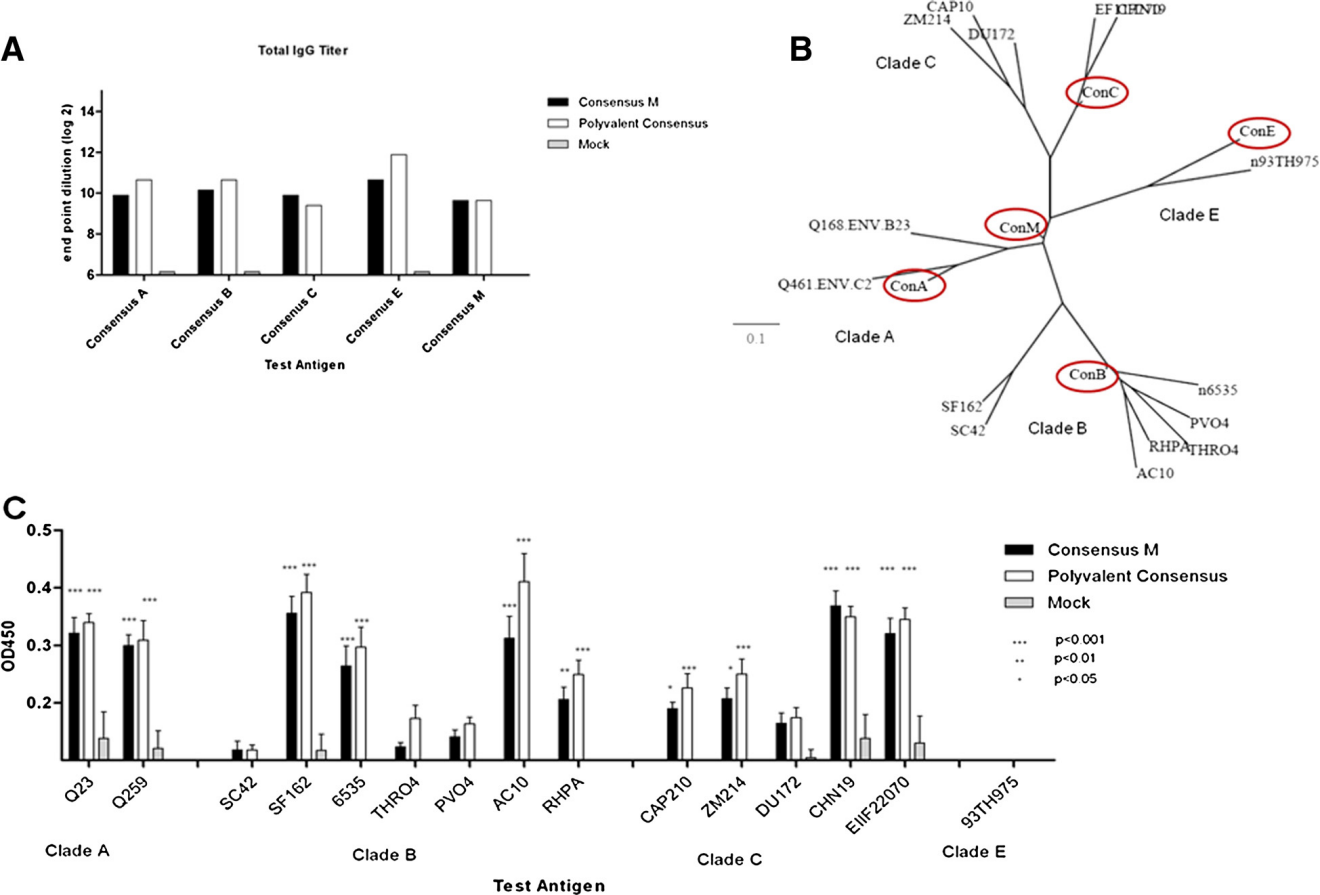
**Binding breadth of vaccinated NHPs sera.** NHP were vaccinated at weeks 0, 4 and 8 with blood collected 14 days after each vaccination. Vaccines were formulated with 300 μg of purified protein and Imject® alum and delivered intramuscularly. **A)** Sera collected on day 35 was pooled and used to determine total IgG via ELISA for each vaccine group. Bar values represent the geometric mean titer (+95% confidence interval) of log2 transformed titers. The endpoint titer is described on the y–axis and the identified Env_gp140_ trimer antigen is described on the x-axis. **B)** The unrooted phylogenetic tree was produced using Phylogeny.fr web service and the 14 HIV-1 Env_gp160_ sequences based upon the list of Env_gp160s_ in Table 2 showing. The Env_gp160s_ were from clades A, B, C, E from 1993-2005. The clades are indicated on the tree and the consensus Env_gp160s_ are circled. **C)** At day 35 post-vaccination, anti-Env_gp160_ IgG was detected in the serum samples (1:100 dilution) against a panel of primary Env_gp120s_ from clades A, B, C and E via ELISA. Bar values represent the geometric mean titer (95% confidence interval) at an OD450. The OD450 values are displayed on the y-axis and the Env_gp120s_ used are listed on the x-axis. A two-way ANOVA with Bonferroni’s post-test was used to evaluate Statistical significance between the vaccines for each test antigen. A *p*-value of less than 0.05 was considered significant.

To determine the ability of the vaccines to induce a protective response in non-human primates, rhesus macaques were vaccinated intramuscularly with 300 μg of total Env_gp140_ protein either Con M or the polyvalent Con Mixture (equal amounts of each Env_gp140_ protein). Animals were treated according to the guidelines of the IACUC of the University of Pittsburgh. All the protocols used were approved by the IACUC of the University of Pittsburgh (#1002617). Monkeys were administered 3 vaccinations of Env_gp140_ formulated with Imject® alum adjuvant. Mock vaccinated monkeys were administered PBS formulated with adjuvant (Table 1). Monkeys vaccinated with Con M or polyvalent consensus developed anti-Env_gp140_ antibody titers greater than 1:400 dilution against all consensus Env_gp160s_ following the three vaccinations (Figure 2A). Collected sera were tested for the ability to bind to a set of primary Env_gp160s_ representing clades A, B, C, and E (Table 2). The diversity of the Env_gp160s_ chosen, as well as their similarity to the consensus Env_gp160s_, is displayed in the phylogenetic tree where Env_gp160s_ cluster into their identified clades (Figure 2B). The Con M did not cluster with any one clade, but as expected, was located in a more central position on the tree.

**Table 1:** Vaccine groups and regimen of non-human primate study

**Vaccine group**	**Animal numbers**	**Vaccine given**	**Other treatment**
Group 1	N1,N2,N3,N4	Adjuvant only	
Group 2	P1,P2,P3,P4	Mixture of Con A,B,C,E Env gp140	
Group 3	M1,M2,M3,M4	Con M Env gp140	Depletion of CD8+ T cells

Each animal was vaccinated 3 times week 0, 4 and 8. Each vaccine was given in combination alum imject© as an adjuvant. The table gives the vaccine groups, the identification number of animals in each group and the vaccine and treatment (in the case of group 3) received by the animals.

**Table 2:** Information of envelopes used for assays

**Env ID**	**Clade**	**Location**	**Mode of transmission**	**Length of infection**	**Mo/yr isolated**	**Corecepter**
Du172.17	C	South Africa	M-F	12 weeks	Nov-98	R5
ZM214M.PL15	C	Zambia	F-M	<13 weeks	Jul-03	R5
CAP210.2.00.E8	C	South Africa	M-F	5 weeks	May-05	R5
CHN19	C	China				R5
HIV16936-2 EF117270	C	India	F-M	1 week	Nov-00	R5
Q168.ENV.B23	A	Kenya	M-F	1 week		R5
Q461.ENV.C2	A	Kenya	M-F	4 weeks		R5
HIV env 6235 clone 3	B	USA	M-M	6 weeks	Mar-95	R5
PVO clone 4	B	Italy	M-M	4 weeks	Jan-96	R5
pRHPA 4259 clone7	B	USA	M-F	< weeks	Dec-00	R5
pTHRO4156 clone 18	B	USA	M-M	1 week	Aug-00	R5
SC 422661.8	B	Trinidad	F-M	4 weeks	Jan-95	R5
SF162	B	USA				R5
93TH975-15	E	Haiti	F-M	<18 weeks	Jan-93	R5

Primary envelopes from various clade was chosen based to have a diversity of locations and mode of mocusal transmission. Envelopes were used to test the presence of binding and neutralizing antibodies elicited by vaccination of consensus vaccines in non-human primates. SF162 was included because it is the challenge virus envelope.

Both Con M and polyvalent consensus Env_gp140_ vaccines elicited anti- Env_gp140_ antibodies that recognized Env_gp160s_ from clade A, B and C. However, the Env_gp160_ SC42, THRO4, PVO4, (clade B), DU172 (clade C) and 93TH975 (clade E) were not significantly recognized by sera collected from vaccinated animals (Figure 2C). Overall, there was no binding preference of the elicited anti-Env_gp160_ antibodies to primary Env_gp160s_ based on clade, location, or year of Env_gp160_ isolation.

### SHIV_SF162p4_ challenge

**Figure 3:**
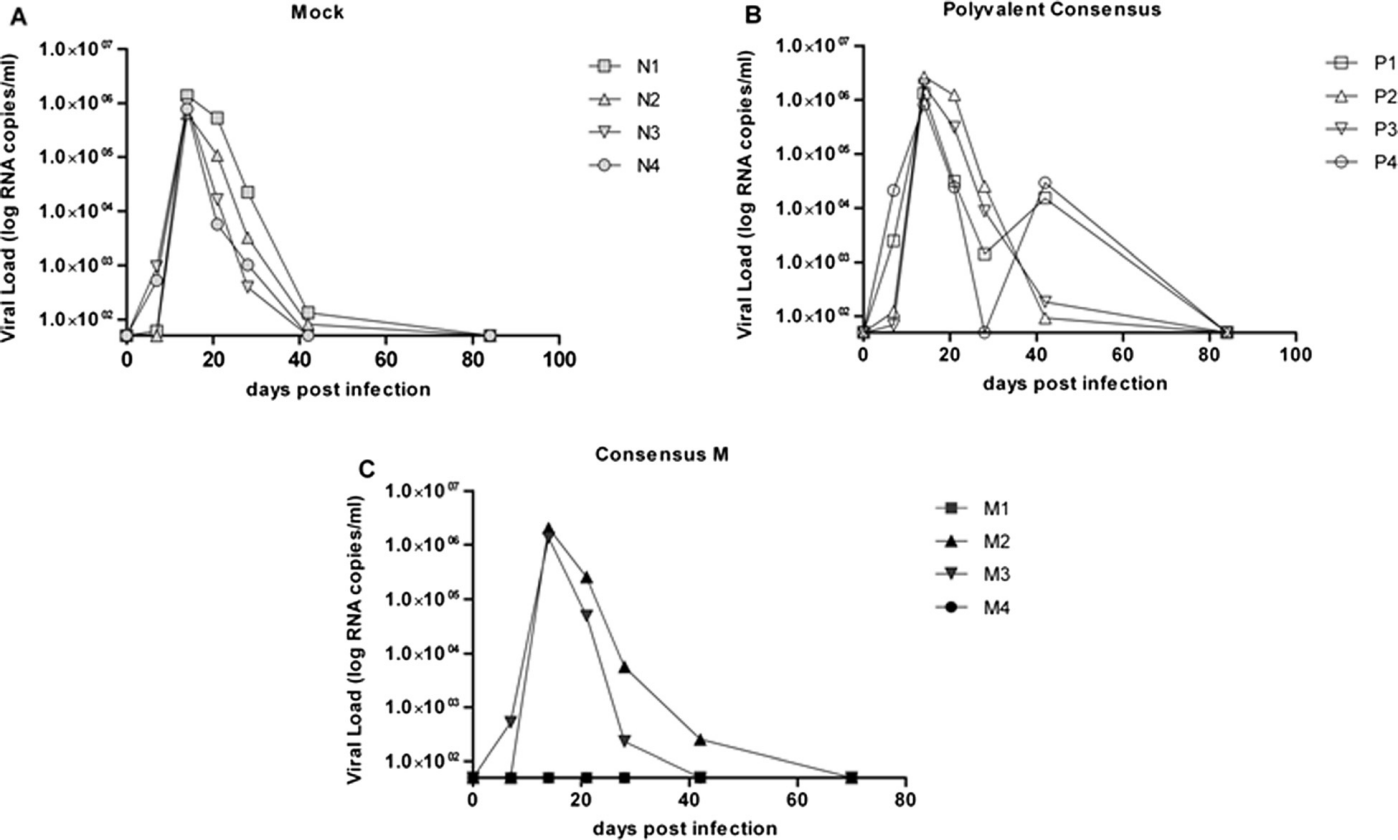
**Heterologous virus challenge.** Monkeys were challenged rectally with 640 TCID_50_ SHIV SF162p4 at week 12 post initial vaccination. Viral titers were determined from collected sera. Viral titers for each individual monkey are displayed as RNA copies/ml of blood on the y-axis by days post challenge on the x-axis. **A)** Mock vaccinated animals (Imject© adjuvant only). **B)** Polyvalent Consensus Env_gp140_ vaccine **C)** Con M Env_gp140_ vaccine.

To evaluate the protective efficacy of each vaccine, monkeys were challenged rectally four weeks after final vaccination with SHIV_SF162p4_ (dose: 640_TCID50_) to ensure all mock vaccinated animals become infected. All mock vaccinated and polyvalent consensus vaccinated monkeys were infected following challenge (Figure 3A and B). Viral loads peaked at day 14 post-challenge at ~1 × 10e+6 RNA copies/ml and then declined to undetectable levels by day 80 post-infection as seen in previous studies using the same stock of virus (unpublished and [8]. Two out of four monkeys (M1 and M4) vaccinated with Con M vaccine had no detectable virus at any time point post-challenge (Figure 3C). Monkeys M2 and M3 had a similar viral pattern as mock vaccinated monkeys with a peak at day 14, followed by a rapid decline.

**Figure 4:**
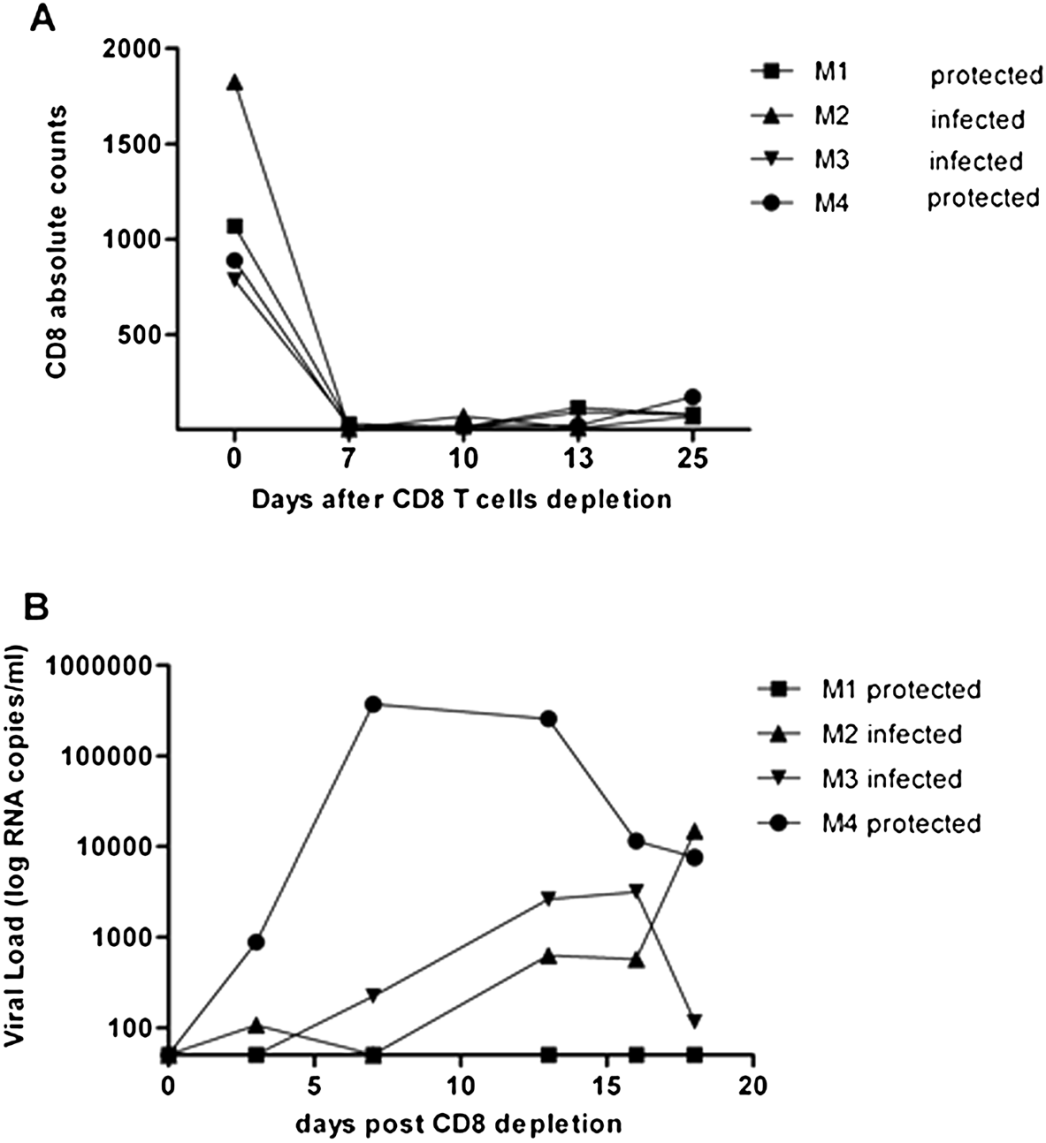
**CD8+ T cell depletion.** Animals vaccinated with Con M Env_gp140_ were all depleted of CD8+ cells by administering the M-T807R1 antibody subcutaneously (50 mg/Kg) at day 0 (day 70 post challenge). **A)** Number of CD8+ cells following antibody administration over the 25 day period of observation. **B)** Viral titers for each individual monkey are displayed as RNA copies/ml of blood on the y-axis by days post challenge on the x-axis.

To determine whether Con M Env_gp140_ vaccinated monkeys were protected from infection, at day 70 post-challenge all Con M Env_gp140_ vaccinated monkeys were depleted of CD8+ cells by administering monoclonal antibody M-T807R1 intravenously [17]. Seven days following antibody administration, no CD8+ cells were detected in the peripheral blood that was sustained for an additional 18 days (Figure 4A). Previously infected monkeys M2 and M3 had a re-emergence of virus during this CD8+-depletion period (Figure 4B). Monkey M4 who was initially aviremic after challenge had an emergence of virus after CD8+-depletion. In contrast, monkey M1 maintained undetectable viral loads following CD8+ depletion.

### Responses to the challenge envelope SF162

**Figure 5:**
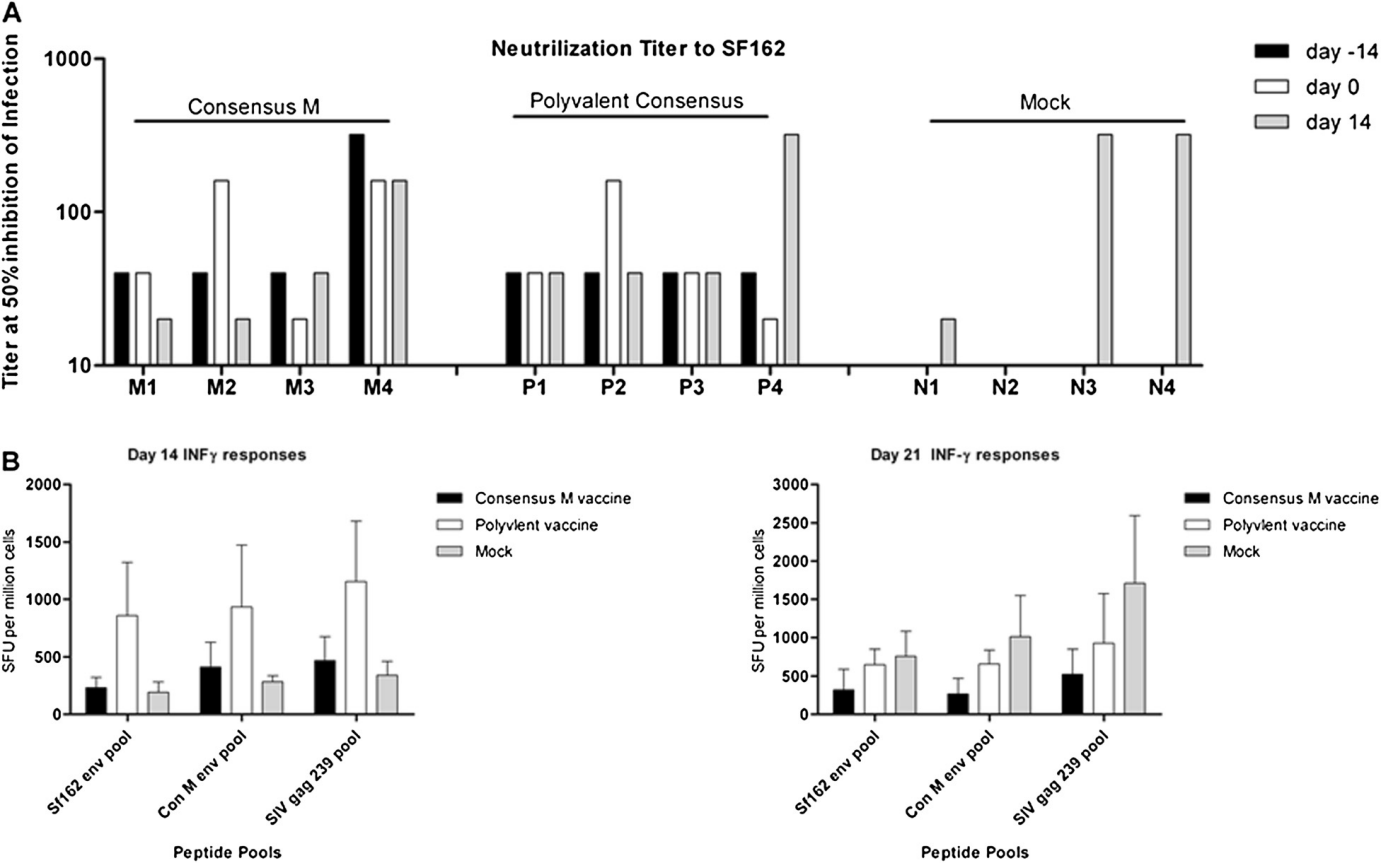
**Neutralizing titers to the SF162 Env**_**gp160 **_**and post challenge cellular responses. A)** Neutralization of HIV_SF162_ by serum collected at day 21 and 35 post-vaccination and 14 post-challenge. The pooled sera dilution at which 50% of virus infection is inhibited is displayed on the y-axis. Neutralizing titers for individuals monkeys are listed on the x-axis by each vaccine group. **B)** INF-γ ELISPOTs were performed using monkey PBMCs collected 14 (left panel) and 21 (right panel) days post-challenge. 1x10^5^ PBMCs were stimulated with 30 ug of Gag peptide pools representing SIV_mac239_ or Env_gp160_ pools representing either Con M or SF162p3 (overlapping peptides, 15-mers with11 amino acid overlap NIH AIDS Research and Reference Reagent program Env 211 peptides and Gag 125 peptides). Spot forming units (SFU) per one million PBMCs are listed on the y-axis and the peptide pools are listed on the x-axis.

At 2 weeks after final vaccination (day -14 prior to challenge), anti-Env_SF162_ IgG antibodies were detected in monkeys vaccinated with either vaccine (Figure 2C). In addition, these antibodies were able to neutralize the ability of HIV_SF162_ to infect cells *in vitro* (Figure 5A). The presence of neutralization titers to all Env_gp160s_ used in the ELISA binding assay were investigated, however, only titers to HIV_SF162_ Env_gp160_ was observed. All vaccinated monkeys had a neutralizing titer of 1:40, except for monkey M4 that had a titer of 1:320. Two weeks following challenge, only monkey P4 had an increase in neutralizing titers. There were detectable neutralization titers in 3 of the 4 mock vaccinated monkeys two weeks post-challenge (Figure 5A). No monkey had antibodies that recognized a set of overlapping SF162 Env_gp160_ peptides. These pools of peptides represented the SF162 Env_gp160_ regions V1/V2, V1 only, or V2 only (data not shown). Interestingly, there was no detectable IFN-γ Env_gp160_ or gag-specific T-cell responses prior to challenge or 7 days post-challenge. At day 14 and 21 post-challenge, IFN-γ T cell responses were detected in all monkeys (Figure 5B), with no significant differences in the number of cells between groups.

## Discussion

HIV-1 Env_gp160_ based vaccines protect monkeys against a homologous SHIV challenges [18,19]. The ability to match the gene sequences used in the vaccine to the possible exposure virus in humans is not possible. Therefore, studies that use a matched Env_gp160_ in the vaccine to the challenge strain is appropriate for proof-of concept studies, but our group set a more challenging goal to protect against a challenge virus with a mismatched vaccine with a limited number of vaccinations. We report here that each consensus sequence representing clade A, B, C, E, in a polyvalent mixture or as a single consensus Env_gp160_ representing the entire Group M elicited anti-Env_gp140_ antibodies that bound to a broad panel of HIV-1 Env_gp160_s. The immunization schedule used was not optimal for antibody affinity maturation; nonetheless, the regimen did induce modest neutralizing antibody titers to the challenge Env_gp160_. However, the elicited immunity did not prevent infection by SHIV_SF162p4_.

In previous studies, consensus sequences designed for clades B and C Gag and Env_gp140_ elicited increased breadth of humoral and cellular immune responses [12,20-22]. Consensus Env_gp160_ sequences representing Group M, termed CON-S, elicited antibodies that neutralized multiple Env_gp160s_, as well as eliciting cross-clade cellular immune responses [13,14]. However, viral challenges of CON-S vaccinated monkeys were not reported and therefore the efficacy of the induced immune response elicited by these vaccines is unknown.

Compared to Env_gp120_ monomers, Env_gp140_ trimers may expose binding and neutralizing epitopes that are present only in Env’s quaternary state [23,24]. Our consensus Env_gp140_ trimers have similar antigenic properties as wild-type Env_gp160s_, as demonstrated by attaching to human CD4 and binding to the monoclonal antibody b12. The b12 antibody recognizes a conserved region on gp120 mapped to a discontinuous epitope overlapping the CD4 binding site [25].

Following three intramuscular vaccinations, all monkeys seroconverted by day 14 following the final vaccination. Nonetheless, there were differences in the vaccine efficacy following challenge between the two vaccine groups. Both non-neutralizing and neutralizing antibodies have been implicated in reducing rates of infection by HIV-1 [26,27]. A report based on the analysis of the sera samples of vaccinated volunteers in the RV144 clinical trial stated that the vaccine elicited antibodies against the V2 region of the HIV-1 Env_gp160_ were correlated with lower rates of HIV infection [28]. Antisera collected from these vaccinated individuals did not neutralize the infection *in vitro*. Additionally, vaccine induced protection against a neutralization resistant virus in macaques was correlated with antibodies to the V2 region of Env_gp160_[29]. Whether antibodies that bind to the V2 region are correlated with protection against SHIV_SF162p4_ infection in this study is unclear. There were no antibodies elicited in monkeys vaccinated with Con M or polyvalent consensus Env_gp140_ vaccines that recognized SF162 Env_gp160_ linear peptides, including those specific to V2 (data not shown). Further studies are necessary to determine if the two protected animals in the Con M Env_gp140_ group elicited antibodies recognizing conformational epitopes, such as the V1/V2 scaffold proteins. The V1/V2 scaffold was used to analyze human sera collected from vaccinated volunteers in the RV144 clinical trial [30,31]. Determining if antibodies specific to various conformational epitopes on Env_gp160_ may explain the differences observed in vaccinated animals following SHIV challenge.

Two monkeys that had no detectable viral levels following SHIV infection were M1 and M4. M2 and M3 had detectable viral levels and therefore were not protected against infection. Monkeys vaccinated with either polyvalent consensus or Con M Env_gp140_ trimers had neutralizing titers to HIV-1_SF162_. Neutralizing antibodies against Env_gp160_ can protect monkeys against viral challenge [32,33]. However, only one monkey (M4) in the present study had high neutralizing antibodies (1:320) against SHIV_SF162p4_ and had undetectable viral titer 14 days after challenge. However, following CD8+ T cell depletion, virus was detected (<1×10^5^ RNA copies /ml) in the blood indicating that infection was not blocked, but may have been controlled by the vaccine elicited antibodies. T cell responses did not appear to play a role in protecting the monkeys from infection. There was no difference in the number or kinetics in the elicitation of Env_gp160_ or Gag specific IFNγ producing cells following challenge in any of the vaccinated monkeys compared to mock vaccinated animals.

Upon CD8+ depletion, it was not unexpected that M2 and M3 had a rebound in blood titer virus, but the detection M4 was unexpected. In contrast to monkey M4, no virus was detected in monkey M1 even after depletion of CD8+ T cells. Both IFNγ specific T cells and neutralizing antibodies were detected, but it is unclear which of these immune responses may have contributed to the protection. In addition, the MHC class I haplotype did not appear to correlate with protection. Even though no viremia was ever detected in monkey M1, it is possible that virus could be located in reservoirs, such as the bone marrow or gut mucosa [34]. The M-T807R1 monoclonal antibody used for CD8+ T cell depletion is specific for cells in the serum and lymph nodes [35], therefore, it may have not depleted cells in reservoirs of hidden virus. In an effort to identify possible reasons for M1 protection the animals’ halotypes were determined. Monkey M1 had a Mamu-B*008 MHC class I haplotype, which has been associated with control of SIV_mac239_ virus; the parent virus of the challenge SHIV_SF162p4_[36]. Therefore, a combination of the neutralizing antibodies, non-neutralizing antibodies and the Mamu-B*008 MHC class I haplotype may have resulted in “sterilizing” protection after viral challenge. However, the Mamu-B*008 MHC class I haplotype was also present in monkey M3, which had similar binding and neutralizing antibody titers as monkey M1, but was not protected from SHIV infection. Interestingly, two monkeys vaccinated with the polyvalent consensus vaccine, P2 and P4, had a rebound in viral titers at day 40 post-infection before returning to undetectable levels (Figure 3B). The rebound virus could have been a variant that escaped the vaccine elicited immune response, however, sequencing of the virus in the blood collected at day 40, did not show any significant variation of the viral sequence compared to the input virus on day 0.

While Env_gp140_ only vaccines have been successful against homologous challenge, both the human RV144 trial and previous monkey studies showed significant protection from heterologous challenge, included other HIV protein components [8,37,38]. Including Tat in the vaccine formulation induces strong and persistent CD4^+^ T cells [39] and broadens T cell responses directed against Gag and Env_gp160_[40,41]. Gag is known for inducing strong cellular responses that may lead to reduced viral loads [42,43]. Addition of Gag and/or Tat to our Con M vaccine may have prevented infection or controlled undetectable virus in vaccinated animals more effectively than Con M Env_gp140_ alone. Even though some of these studies use Env_gp140_ proteins, they are combined with other HIV proteins to elicit a broadly reactive response. In our vaccine presented here, the purified VLPs only have Gag and Env_gp140_ expressed in different modalities than VLPs and we achieve a broadly reactive anti-Env_gp140_ response using our consensus Env_gp160s_. For example, viral vectors are used to express Gag and Env_gp160_ independently in the RV144 human trial, which really does not allow for comparison with our VLP strategy.

## Conclusion

Rhesus macaques were vaccinated with trimerized Env_gp140_ proteins representing consensus sequences for clade A, B, C, E, in a polyvalent mixture or as a single consensus Env_gp160_ representing the entire Group M. These consensus Env_gp140_ elicited antibodies with cross-clade anti-Env_gp140_ binding against a panel of HIV-1 Env_gp160s_. However, this breadth of antibody binding to HIV-1 Env_gp160s_ only partially correlated with the prevention of infection by SHIV_SF162p4_.

## Materials and methods

### Vaccines design

The consensus sequences represent the most common amino acids found at each position of the aligned envelope sequences used. One hundred Env_gp160s_ sequences per clade were used to design consensus sequences of clades A, B, C, and E. The design of the group M consensus Env_gp160_ was based on two hundred Env_gp160_ sequences representing clades A, B, C, D, E, F and H. Env_gp160s_ chosen were isolated following mucosal transmission, within weeks after infection and included a diversity of viruses that were isolated in different locations around the world between 1995 and 2005. Each Env_gp160_ used the CCR5 co-receptor. Vaccine immunogens were designed as Env_gp140_ trimers as previously described [15].

### Protein purification

Human embryonic kidney (HEK) 293T cells were transiently transfected with DNA (8 μg) expressing one of the consensus HIV-1 Env_gp140_ proteins or wild type Env_gp120_ 6X-HIS tagged proteins. Following DNA transfection as previously described [44], secreted Env_gp140_ proteins were purified using lectin columns made from agarose *galanthus nivalis* (snowdrop) lectin (Vector laboratories, Burlingame, CA, USA) [45] and the Env_gp120_ 6X-HIS tagged proteins were purified using nickel columns [44]. Other purified Env_gp160s_ used for ELISAs were purchased from eEnzyme (Gaithersburg, MD, USA). Each purified consensus Env_gp140_ trimer protein (1 μg) was loaded on to NativePAGE native gel (Invitrogen, Carlsbad, CA, USA) and separated by electrophoresis in the manufacture’s recommended buffers. After separation, the proteins were detected using the ProteoSilver Sliver Stain kit (Sigma, St. Louis, MO, USA) following manufacturer’s protocol [44].

### CD4 binding assay

The CD4 binding assay was performed to demonstrate Env_gp160_ binding to its primary receptor using a similar protocol as previously described [46]. Protein G Dynabeads (Invitrogen) were mixed with anti-his antibody. The tubes with the mixture were placed on the magnets to remove all unbound antibody. Then soluble human CD4-6XHIS tagged protein (eEnzyme, Gaithersburg, MD, USA) and consensus Env_gp140_ mixtures were then mixed with the beads. Following pellet fractionation, samples were separated on a 10% SDS PAGE gel, transferred unto a nitrocellulose membrane and probed for sCD4 or Env_gp160_ using specific antibodies. Following secondary IgG-HRP antibodies which were used to detect proteins by Western blot.

### Surface plasmon resonance

MAb b12 binding kinetic analyses of the HIV rgp140(s) was performed by surface plasmon resonance (SPR) on a Biacore 3000 (GE/Biacore AB, Inc., Uppsala, Sweden) as previously described [47].

### Animals and vaccination

Animals were treated according to the guidelines of the IACUC of the University of Pittsburgh. All the protocols used were approved by the IACUC of the University of Pittsburgh (#1002617). Rhesus macaques (*Macaca mulatta*) were used for all non-human primate experiments. All animals were cared for adhering to USDA guidelines for laboratory animals. Rhesus macaques were anesthetized using 10-20 mg/kg ketamine and vaccinated intramuscularly in the quadriceps and formulated with Imject® alum adjuvant (Imject® Alum, Pierce Biotechnology; Rockford, IL, USA). Vaccinations were completed at weeks 0, 4 and 8. Twelve animals were divided into three (3) groups, four animals per group (Table 1). For blood sample collection animals were anesthetized with a mixture of ketamine/xylazine. Sera was then harvested and stored at -80°C until needed.

### Immune assays

Total anti-Env_gp160_ IgG was detected by enzyme-linked immunosorbent assay (ELISA) using Concanavalin A (50 μg/μl) per well as previously described [8]. End point titer for assay was determined as the reciprocal of the dilution at which the optical density reading was above the mean plus two standard deviations of naïve sera. For *In vitro* neutralization, antisera were tested for the ability to neutralize virus infection *in vitro* using TZM-Bl indicator cells [8]. The sera dilution necessary to neutralize virus was calculated by the following formula (relative light units (RLU) of virus only-RLU of cell only)/2 + RLU cell only. For assessment of T cell responses, NHP IFN-γ ELISPOT was used to enumerate anti-Env_gp160_ specific cellular responses. The number of anti-Env_gp160_ (SF162p3 and Con M) and Gag (SIV_mac239_) specific IFN-γ secreting cells were determined using the non-human primate enzyme-linked immunospot (ELISPOT) assay (R&D Systems, Minneapolis, MN, USA).

### SHIV viral load determination

Real time PCR-based SIV viral detection assay was used to determine the viral titers post-challenge as described in [48]. cDNA (10 μl) generated by the RT-RCR reaction was then used for PCR using the ABI 7000 Gene detection system (Applied Bioscience, Carlsbad, California, USA).

### Anti-CD8 cell depletion by antibody administration

All animals in the Con M group were depleted of CD8+ T cells. The antibody M-T807R1 (NIH NHP Reagent Source, Beth Israel Deacones Medical Center, Boston, MA, USA) was administered subcutaneously (50 mg/Kg) on day 0 (Day 70 post infection). CD8+ T cell depletion was verified using TruCOUNT tubes (BD Bioscience, San Jose, CA, USA).

### Statistical analysis

Statistical tests were performed using Graph Pad Prism software. Statistical significance of antibody test was determined by two-way analysis of variance (ANOVA) followed by the Bonferroni’s post-hoc test. Post-test was used to analyze differences between the vaccine groups. Significance was determined to be a p<0.05.

## Competing interests

The authors declare that they have no competing interests.

## Authors’ contributions

HSE designed and performed most of the experiments analyze data and wrote the paper. BRP was instrumental in animal care and processing of animal samples. JKC performed and analyzed all Surface plasmon resonance data. She was also involve in discussion on results and implications and commented on the manuscript. Finally TMR was the supervisor on the project and was involved in discussions on the results and its implications. He was also instrumental in editing of the manuscript at all stages. All authors read and approved the final manuscript.

## Supplementary Material

Additional file 1: Figure S1 Representative sensograms of IgG b12 SPR data. SPR binding isotherms detailing the interaction of each recombinant trimeric Env_gp140_ with the monoclonal antibody b12 are displayed. Indicated concentrations of each Env_gp140_ were flowed over captured b12 at 37°C on CM5 chips as detailed in Materials and Methods. Association rates, dissociation rates, and affinity constants were calculated with BIA evaluation 4.1.1 software (GE/Biacore AB, Uppsala, Sweden). **A**, ConA; **B**, ConB; **C**, ConC; **D**, Con M; **E**, ConE; **F**, ADA; **G**, PV0.04; **H**, R2; **I**, SC42; and **J**, YU2. Black lines are determined 1:1 Langmuir kinetic fits. RU, resonance units. Click here for file
