# Supplementary figures and images for: Rhesus macaques vaccinated with consensus envelopes elicit partially protective immune responses against SHIV SF162p4 challenge
Source: Virol J. 2013 Apr 2;10:102. doi: 10.1186/1743-422X-10-102 (PMC3637437; doi:10.1186/1743-422X-10-102)

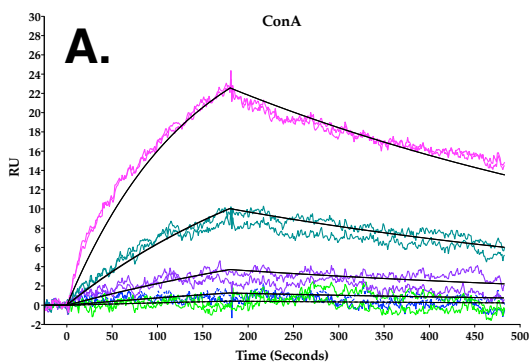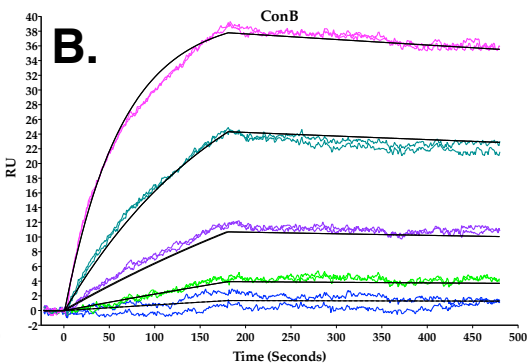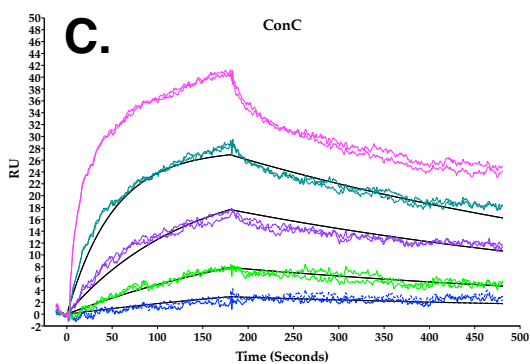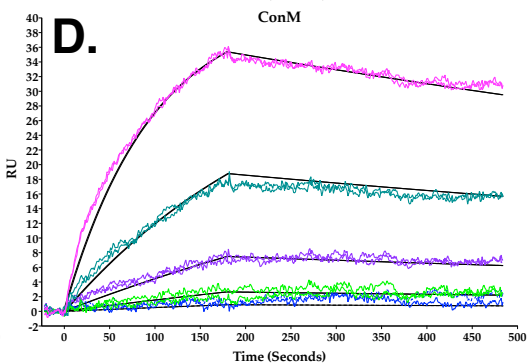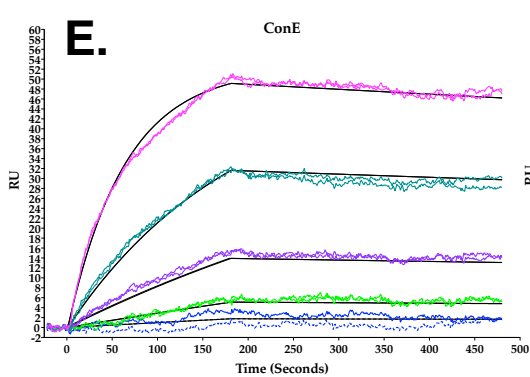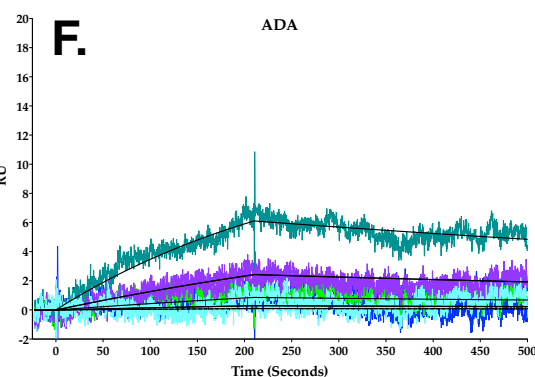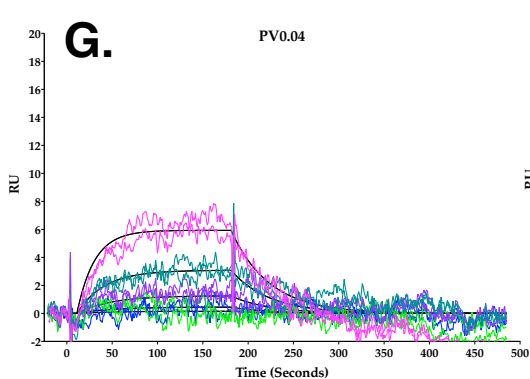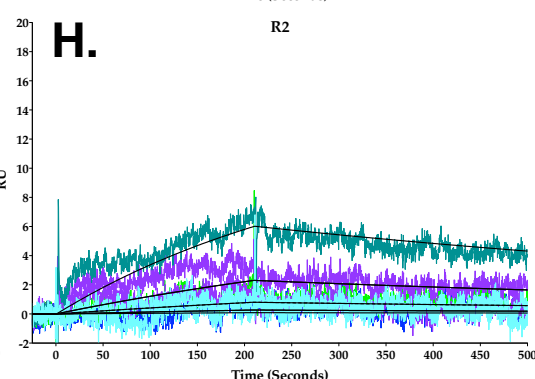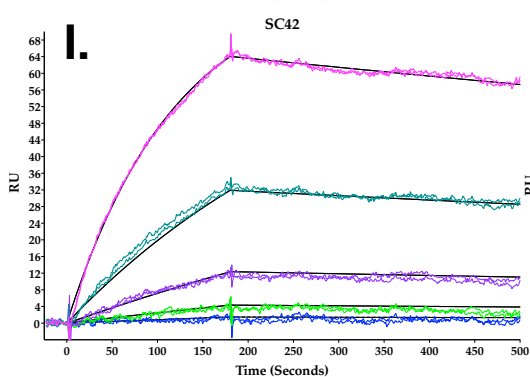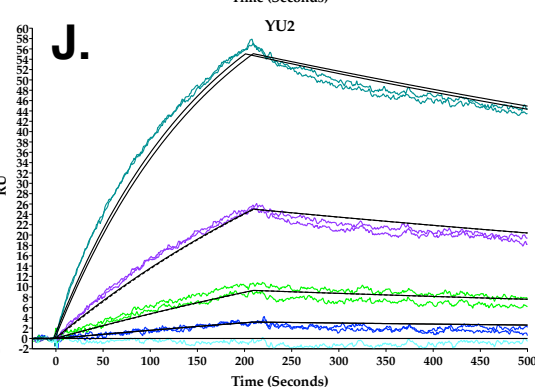

— 0.272nM — 0.815nM — 2.44nM — 7.33nM — 22nM — 66nM

Supplement: Additional file 1: Figure S1 — Representative sensograms of IgG b12 SPR data. SPR binding isotherms detailing the interaction of each recombinant trimeric Envgp140 with the monoclonal antibody b12 are displayed. Indicated concentrations of each Envgp140 were flowed over captured b12 at 37°C on CM5 chips as detailed in Materials and Methods. Association rates, dissociation rates, and affinity constants were calculated with BIA evaluation 4.1.1 software (GE/Biacore AB, Uppsala, Sweden). A, ConA; B, ConB; C, ConC; D, Con M; E, ConE; F, ADA; G, PV0.04; H, R2; I, SC42; and J, YU2. Black lines are determined 1:1 Langmuir kinetic fits. RU, resonance units. [file 1743-422X-10-102-S1.pdf]
